# Photobiomodulation (660 nm) therapy reduces oxidative stress and induces BDNF expression in the hippocampus

**DOI:** 10.1038/s41598-019-46490-4

**Published:** 2019-07-12

**Authors:** Jin-Chul Heo, Ji-Ae Park, Dae-Kwang Kim, Jong-Ha Lee

**Affiliations:** 1 Department of Biomedical Engineering, School of Medicine, Keimyung University, Daegu, 42601 Korea; 2 Department of Medical Genetics, Hanvit Institution for Medical Genetics, Keimyung University, Daegu, 42601 Korea

**Keywords:** Cell death in the nervous system, Cognitive neuroscience

## Abstract

Photobiomodulation therapy (PBMT) effects an important role in neural regeneration and function enhancement, such as expression of nerve growth factor and nerve regeneration, in neuronal tissues, and inhibition of cell death by amyloid beta in neurons is inhibited by PBMT. However, there no studies evaluated the effects of PBMT on oxidative stress in the hippocampus. The aim of this study is to evaluate the effects of PBMT on oxidative stress in the hippocampus. This study assessed the anti-oxidative effect, the expression of BDNF and antioxidant enzymes, as well as the activation of cAMP response element binding (CREB) and extracellular signal-regulated kinase (ERK) signal transduction pathways assess using a hippocampal cell line (HT-22) and mouse organotypic hippocampal tissues by PBMT (LED, 660 nm, 20 mW/cm^2^). PBMT inhibited HT-22 cell death by oxidative stress and increased BDNF expression via ERK and CREB signaling pathway activation. In addition, PBMT increased BDNF expression in hippocampal organotypic slices and the levels of phosphorylated ERK and CREB, which were reduced by oxidative stress, as well as the expression of the antioxidant enzyme superoxide dismutase. These data demonstrate that PBMT inhibits hippocampal damage induced by oxidative stress and increases the expression of BDNF, which can be used as an alternative to treat a variety of related disorders that lead to nerve damage. Activation and redox homeostasis in neuronal cells may be a notable mechanism of the 660-nm PBMT-mediated photobioreactivity.

## Introduction

Brain-derived neurotrophic factor (BDNF) effects on specific neurons in the central and peripheral nervous systems to promote growth and survival and differentiation of new neurons and synapses^1,2^. BDNF is known to have a variety of effects in the nervous system. In the brain, it is expressed in the hippocampus, cortex, and basal brain and is involved in learning and memory^3^. BDNF is important for long-term memory and helps stimulate and regulate nerve development. During stress exposure, corticosterone appears to reduce the expression of BDNF in rats, and sustained stress causes atrophy in the hippocampus^4–6^. It has been shown that atrophy in the hippocampus and other limbic systems occurs in people with depression^7^. In a rat model of induced post-traumatic stress disorder, treatment with the antidepressant fluoxetine was shown to increase BDNF expression, as well as exercise capacity^8^. Moreover, increased expression of BDNF in a genetic model of cognitive deficits leads to improved social and cognitive function^9^. In addition, the expression of BDNF is reduced in an amyloid-β1-42 (Aβ1-42)-induced Alzheimer’s rat model, while BDNF administration was shown to increase cognitive function^10^. As such, BDNF acts on the nervous system and exhibits excellent effects for the treatment of related diseases.

Photobiomodulation therapy (PBMT), a treatment method using infrared or near-infrared light (600–1100 nm), is used in traumatic and degenerative brain diseases^11,12^. PBMT (808 nm) irradiation could help cerebrospinal fluid (CSF) to induce neuronal differentiation of human umbilical cord mesenchymal stem cells (hUC-MSCs) in early stage, and can enhance the mesenchymal stem cell (MSC) tissue repair, differentiation and proliferation^13^. Photoreceptor regulation of cytochrome c oxidase activity is the most important mechanism of action, and PBMT can enhance secondary metabolism by modulating mitochondrial function, neurotransmission, and redox status^14^. This therapy is based on the activation of signaling pathways by photons, in response to specific molecules in living organisms. Recently, PBMT has been clinically applied for a wide range of medical indications, including wound healing promotion; reduction of pain, edema, and inflammation caused by cell and tissue death; and differentiation and proliferation effects^15–18^.

Various studies have been carried out to increase the expression of BDNF using light. For example, nano-pulsed laser therapy was applied in rats to show that BDNF expression can be increased by a noninvasive method^19,20^. Moreover, PBMT increased the expression of BDNF in amyloid beta-treated neurons and hippocampal cells, thus inhibiting neuronal damage and inducing dendritic cell activity^21^. However, there are still many uncertainties regarding the efficacy and mechanism underlying PBMT effects using visible light sources to treat brain damage. Unlike chemical compounds and proteins, it is not easy to identify signaling pathways activated by light wavelengths in specific areas. Phototherapy possibly works through the activation of a photoreceptor system, and the mitochondrial electron transport chain is known to be sensitive to infrared and near infrared light^22^. However, when applied to diseases, it is not easy to find proteins that are specifically targeted by light.

In this study, we assessed whether, similarly to PBMT, a 660-nm LED therapy reduces hippocampal cell damage. Specifically, we examined PBMT effects under oxidative stress, the expression of antioxidant enzymes, as well as BDNF expression and related signaling pathways in hippocampal cells and tissue.

## Results

### LED at 660 nm inhibits cell death by reducing oxidative stress

**Figure 1:**
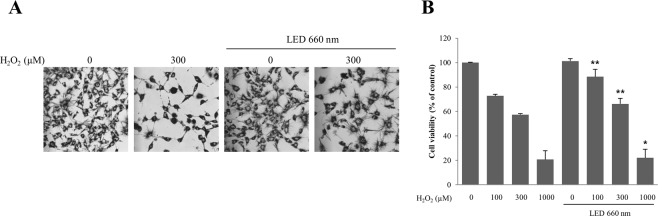
LED at 660 nm inhibits intracellular oxidative stress. (**A**,**B**) HT-22 hippocampal cells were subjected to oxidative stress induced by hydrogen peroxide (H_2_O_2_, 300 μM) with or without LED at 660 nm, as indicated. After incubation, cell morphology was assessed using phase contrast microscopy (**A**) or the MTT assay (**B**). The relative cell viability of LED-treated HT-22 cells is shown in B. NT, not treated. *p < 0.05 and **p < 0.01 vs control.

The hippocampal HT-22 cell line was used to assess the effects of H_2_O_2_-induced oxidative stress on cell survival and the cell death-suppressing effect by the 660-nm LED (Fig. 1A). After treatment of HT-22 cells with 100, 300, and 1000 μM H_2_O_2_, the cell viability was 72.7, 57.3, and 20.7%, respectively. In contrast, treatment with the 660-nm LED increased the cell viability to 88.4, 66.2, and 22.0%, respectively. The percent increase in cell viability in the presence of H_2_O_2_ at 100, 300, and 1000 µM was 15.7, 8.9, and 1.3%, respectively (Fig. 1B). The results show that the 660-nm LED suppresses the oxidative stress caused by H_2_O_2_, which increases the survival rate of cells exposed to oxidative stress.

### LED at 660 nm increases BDNF expression in hippocampal cells through the activation of ERK and CREB signaling pathways

**Figure 2:**
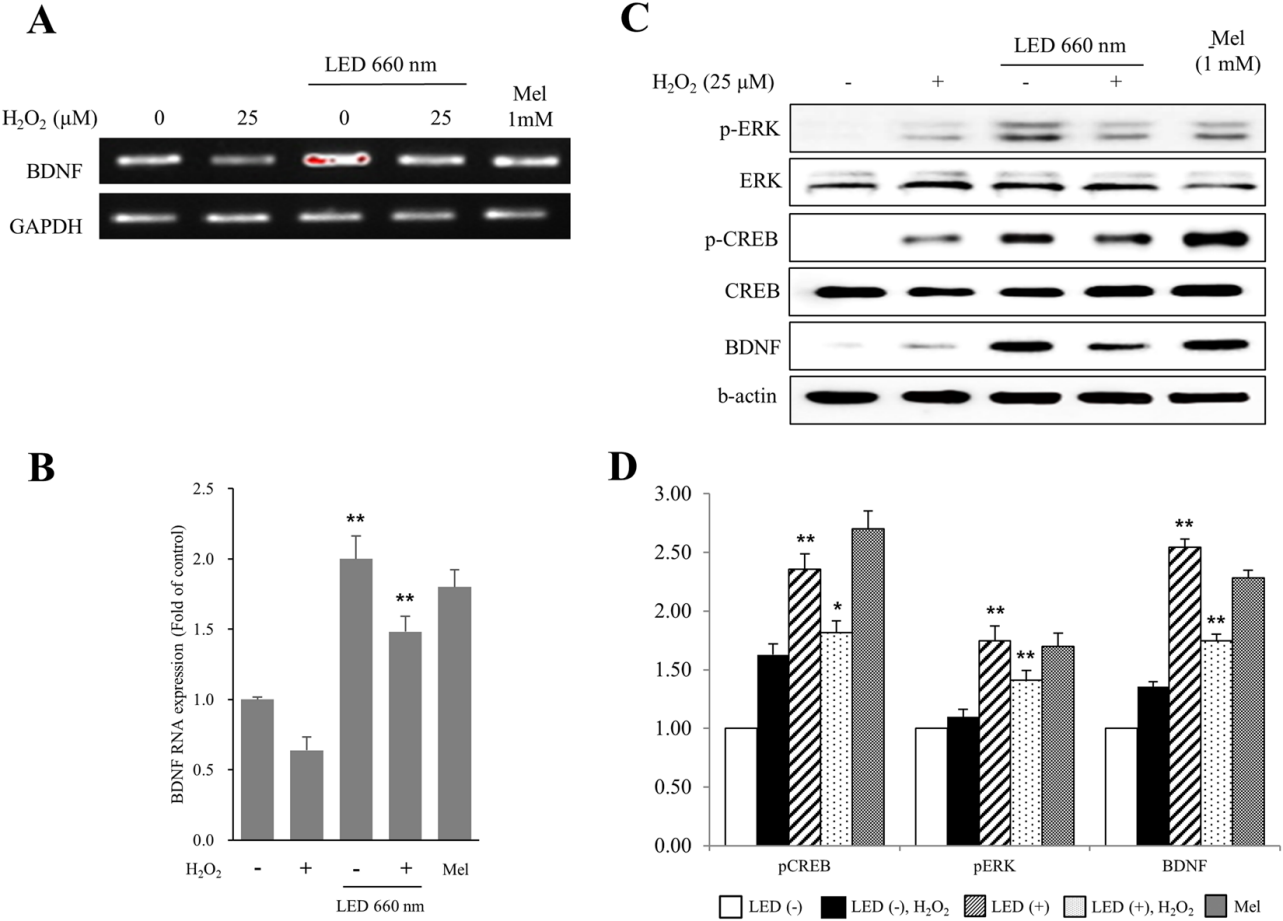
LED irradiation of HT-22 cells at 660 nm increases the expression of *BDNF* through the ERK and CREB signaling pathways. HT-22 cells were subjected to oxidative stress by H_2_O_2_ (25 μM), and mRNA expression was assessed by RT-PCR (**A**,**B**) and western blot (**C**,**D**). The expression of BDNF mRNA and protein levels were increased by the LED light. Melatonin (Mel) treatment (1 mM) was used as a positive control. (**B**) Quantification of *BDNF* expression levels in LED-treated HT-22 hippocampal cells relative to *GAPDH* levels. (**C**,**D**) Western blot analysis to examine the activation of ERK and CREB signal transduction pathways by LED. Antibodies used are as indicated. The expression of BDNF increased presumably due to the increased activation of ERK and CREB by LED, indicated by the enhanced levels of p-ERK and p-CREB. (**D**) Quantification of protein levels in LED-treated HT-22 hippocampal cells relative to GAPDH, used as a loading control. NT, not treated. *p < 0.05 and **p < 0.01 vs control.

The expression of BDNF in HT-22 cells was assessed by RT-PCR. Irradiation of HT-22 cells with the 660-nm LED increased BDNF expression by about 2-fold. Although H_2_O_2_ treatment reduced BDNF expression, LED irradiation in the presence of H_2_O_2_ could still increase BDNF expression by about 2.1-fold. Melatonin at 1 mM was used as a positive control (Fig. 2A,B).

In order to investigate the signal transduction pathway leading to BDNF upregulation, we examined ERK and CREB activation by assessing their phosphorylation levels. We found that phosphorylation of ERK and CREB was increased upon irradiation with the 660-nm LED light. H_2_O_2_ treatment increased p-ERK and p-CREB levels, which were further increased upon LED irradiation at 660 nm (Fig. 2C,D). These results suggest that the ERK and CREB signal transduction pathways might mediate the increased BDNF expression in HT-22 cells following 660-nm LED irradiation.

### LED at 660 nm increases the expression of BDNF in the mouse hippocampus

**Figure 3:**
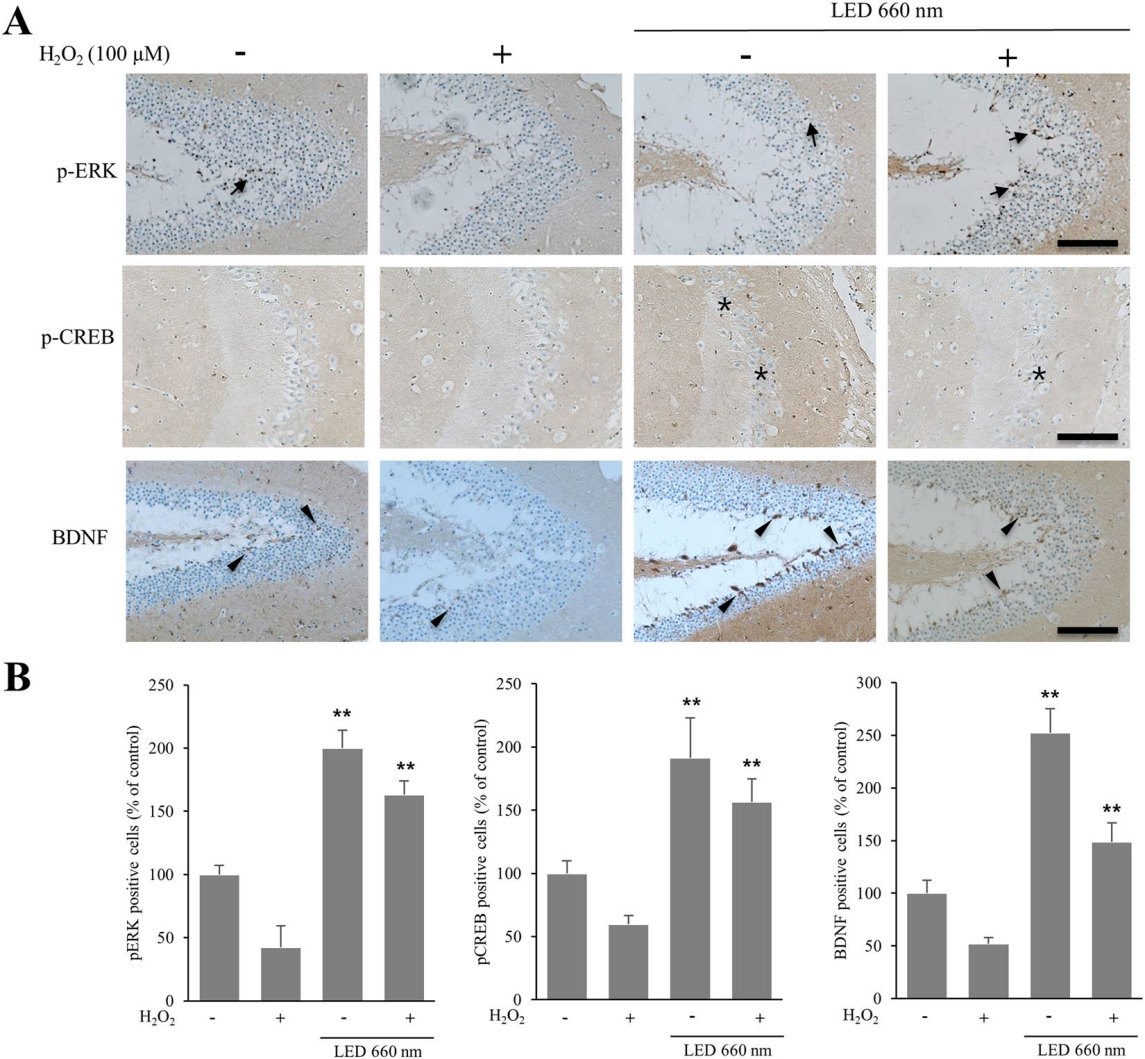
LED at 660 nm enhances the expression of BDNF in the hippocampus. Hippocampal organotypic slice cultures were subjected to oxidative stress by H_2_O_2_ (100 μM), and irradiated with LED at 660 nm. (**A**) Representative sections of organotypic slices stained with antibodies against p-ERK, p-CREB, and BDNF, as indicated, and counterstained for hematoxylin/eosin. Arrows, asterisks, and arrowheads indicate positively immunostained cells. (**B**) Quantification of p-ERK-, p-CREB- and BDNF-positive cells in LED-treated hippocampal slices relative to control. The graph shows the percentage of immunostained positive cells when compared to the cells of control hippocampal tissue in which oxidative stress was not induced. NT, not treated; plus and minus symbols indicate the presence or absence of H_2_O_2_. **p < 0.01 vs control. Scale bar = 100 µm.

BDNF upregulation by LED irradiation was confirmed in the mouse hippocampus by immunohistochemistry. The number of BDNF-expressing cells was 2.5 and 2.8 times higher in the case of LED treatment and H_2_O_2_ treatment, respectively, of mouse hippocampal organotypic slice cultures (Fig. 3A,B). We also found that the number of p-ERK- and p-CREB-positive cells was higher with than without LED irradiation. Upon LED irradiation, p-ERK-positive cells were increased by about 2- and 3.8-fold without or with H_2_O_2_, respectively. Under the same conditions, p-CREB was increased by about 1.6- and 3.3-fold, respectively. These results support that the increased levels of BDNF by LED in the hippocampus might be mediated by ERK and CREB signaling pathways.

### LED at 660 nm promotes the activity of antioxidant enzymes in the hippocampus

**Figure 4:**
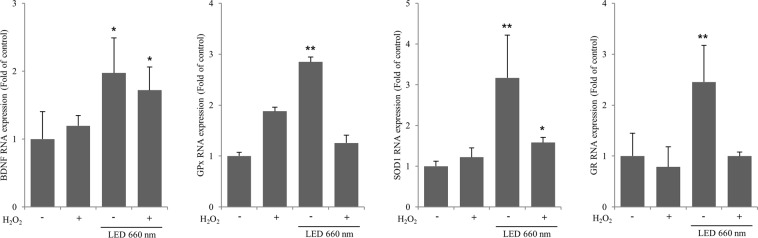
LED at 660 nm enhances the activity of the antioxidant enzymes glutathione peroxidase (GPx), superoxide dismutase (SOD1), and glutathione reductase (GR). mRNA levels of antioxidant enzymes in organotypic hippocampal slices were examined by RT-qPCR after induction of oxidative stress by H_2_O_2_ (100 μM) and LED irradiation. Values represent the fold-change in expression levels compared to control. The expression of GPx, SOD1, and GR was increased according to the increase in BDNF expression. NT, not treated. *p < 0.05 and **p < 0.01 vs control.

We examined the mRNA expression of BDNF and of the antioxidant enzymes glutathione peroxidase (GPx), superoxide dismutase 1 (SOD1), and glutathione reductase (GR) in mouse hippocampal organotypic slice cultures irradiated with LED at 660 nm (Fig. 4). BDNF expression was increased in both control and H_2_O_2_-treated hippocampal slices upon LED irradiation. GPx expression was increased by approximately 2.7-fold by LED irradiation. However, expression was decreased after H_2_O_2_-induced oxidative stress. LED irradiation also increased SOD1 and *GR* levels by about 3- and 2.4-fold in control slices and SOD1 levels by about 1.6-fold in the H_2_O_2_-treated slices; in the latter condition, LED did not affect GR levels. These results show that LED at 660 nm induces BDNF expression in the hippocampus, as well as the expression of the antioxidant enzymes GPx, SOD1, and GR. In addition, the 660-nm LED increased the expression of SOD1 upon induction of oxidative stress by H_2_O_2_.

## Discussion

In our study, we demonstrated that 660 nm inhibited apoptosis induced by oxidative stress in hippocampal cell line and increased BDNF expression. Consistently, when wheat and soybean seeds were treated with LED (638 ~ 731 nm), the total phenol content and the anti-oxidative properties of α-tocopherol and vitamin C were significantly increased^23^. PBMT on ischemia-reperfusion injury in the abdominal muscle of rats increased the activity of antioxidants^24^.

Phototherapy by PBMT is based on the absorption of a photon produced by a LED light source by photoreceptors present in tissues and promotes cellular metabolism^25,26^. Light absorbed by cells increases reactive oxygen species (ROS) production and ATP synthesis in tissues^22^. ROS are associated with gene expression^27^. In addition, cells exposed to red and near-infrared light produce nitrogen oxide (NO). At the appropriate time and frequency, light of a selected wavelength can be absorbed by a photoreceptor, such as the intracellular cytochrome c oxidase. This promotes NO photolysis, hsp-70i enzyme activation, mitochondrial metabolism, and ATP production^28^. As a result, the overall metabolic activity of the cell is actively induced. Although under normal conditions PBMT increases ROS, under oxidative stress, it reduces high ROS levels and inhibits the apoptosis of cortical neurons^29^, while combination of PBMT in wound healing stimulates the reduction of ROS and activates ERK1/2 to promote wound healing^30^. Consistently, in our cell line-based experiments, we confirmed that the 660-nm LED suppresses the oxidative stress caused by H_2_O_2_. Interestingly, we found that the cell survival rate was higher under oxidative stress induced by a low than by a high H_2_O_2_ concentration. This indicates that the inhibitory effect of the 660-nm LED on oxidative stress is exerted by reducing oxidative stress at low H_2_O_2_ concentrations.

In our study, we found that the 660-nm LED increases the expression of BDNF via the phosphorylation of ERK and CREB. The bio-stimulatory effects of PBMT generally promote cell survival and proliferation and can activate redox sensitive signaling pathways, such as those mediated by nuclear factor E2-related factor 2 (NRF2), nuclear factor-κB (NF-κB), and ERK, which serve as redox checkpoints^25^. A previous study showed that BDNF expression is reduced by inhibition of ERK in an amyloid-β1-42-induced Alzheimer’s model in rats, and that increased BDNF expression in the hippocampus through activation of the ERK signaling pathway prevents learning disorders^10^. Moreover, PBMT at 632.8 nm was shown to increase BDNF expression through the activation of the ERK/CREB pathway, thus inhibiting Aβ-induced nerve damage^21^. Although the activity of ERK and CREB is known to promote BDNF expression, our study confirmed that LED light at 660 nm exerts its effects by these pathways by oxidative stress.

The main hypothesis to explain the effects of PBMT is that cytochrome c oxidase (unit IV in the mitochondrial respiratory chain) absorbs light into the near-infra-red region.by heme and copper centers. Photon increases the electron transport, mitochondrial membrane potential, and ATP resulting from the dissociation inhibitory nitrogen oxides in enzyme, finally inducing the activation of a light-sensitive ion channel that allows calcium to enter the cell. After light absorption, the upregulation of ROS, cyclic AMP, NO, and Ca^2+^ activate numerous signaling pathways leading to the activation of transcription factors that in turn increase the expression of antioxidant enzyme-related genes^26^. In this study, we confirmed that the expression of the antioxidant enzymes GPx, SOD1, and GR in hippocampal slices irradiated with 660 nm was also increased.

The limitation of this study is that the output of the 660-nm LED was used only at 20 mW/cm^2^. Thus, the power was very low and had a small effect on cells and tissue. Moreover, we could not assess the effects using various outputs, so the optimal conditions for enhancing antioxidation could not be confirmed in the present study system.

Although the development of new drugs using natural products or compounds has been difficult due to safety and cost of long-term development, low power phototherapy is relatively safe and has been used for various diseases for a long time. It is expected that the application range of phototherapy in disease treatment will gradually increase, and future studies should identify the related cell signaling pathways.

## Materials and Methods

### Animals

A total of 7 healthy seven-weeks-old C57BL/6 mice (Hyochang Science, Korea) were placed in individual cages and maintained a light/dark cycle of 12 hours using food and water as needed. Every effort has been made to minimize the number of animals used and to limit animal suffering. Animals were anesthetized with 5% isoflurane (JW Pharm, Korea). Anesthesia was maintained throughout the surgical procedure. All procedures were carried out in accordance with the animal welfare committee ‘s guidelines on Guiding Principles in the Care and Use of Animals National Research Council 1996. The Institutional Animal Care and Use Committee (IACUC) approval number for Keimyung University was KM201743R2.

### Antibodies and chemicals

Antibodies against phosphorylated-cAMP response element binding (CREB) protein (p-CREB; Ser133), and CREB (48H2) were obtained from Cell Signaling Technology, Inc. (Beverly, MA, USA); against p-extracellular signal-regulated kinases (ERK) 1/2 (Thr202/Tyr204), ERK1/2 (MK1) and BDNF (N-20) were purchased from Santa Cruz Biotechnology (Santa Cruz, CA, USA). A monoclonal anti-β-actin antibody, methylthiazolyldiphenyl-tetrazolium bromide (MTT) and melatonin were provided by Sigma-Aldrich (St. Louis, MO, USA).

### Light treatment

A LED-based device prototype equipment was used as light source in the experiments. The experimental groups were irradiated with 660-nm with a power density of 20 mW/cm^2^ (LED4D067, Thorlabs Inc., Newton, New Jersey, United States). The total energy densities delivered were 3 J/cm^2^. All parameters of the laser device were selected in Table 1.

**Table 1:** Irradiation parameters.

Paremeter [unit]	Value
Center wavelength [nm]	660
Output mode	Continue
Average radiant power [mW]	1000
Spot area [cm^2^]	20
Irradiance at aperture [mW/cm^2^]	20
Beam profile	Round
Beam divergence	90
Spectral bandwidth [nm]	25
Frequency [Hz]	2.5

### Hippocampus cell line culture

The mouse hippocampal neuronal cell line HT-22 was grown in Dulbecco’s modified Eagle’s medium (DMEM; Gibco/Invitrogen, Grand Island, NY) supplemented with 10% heat-inactivated fetal bovine serum (Gibco/Invitrogen), 0.01% penicillin/streptomycin (Gibco/Invitrogen), at 37 °C in a humidified 5% CO_2_ incubator.

### MTT assay

Protection against cell death was assayed using the CCK-8 kit (Dojindo, Gaithersburg, MD) and MTT, as follows: HT-22 cells (5 × 10^5^/mL) were plated in 96-well plates, and incubated for 24 h in 100 μL of DMEM medium. Various concentrations of H_2_O_2_ were added to the cells, and cells were incubated for an additional 24 h with a 660-nm LED irradiation. Next, 10 μL of MTT solution (5 mg/mL MTT in PBS) was added to each well, followed by incubation at 37 °C for 2 h. Absorbance was measured with a Victor multi label counter (Wallac, Turku, Finland) at 450 nm and 564 nm^31^.

### Preparation of organotypic hippocampal slice cultures

Organotypic hippocampal slice cultures (OHCs) were prepared under sterile conditions using a slightly modified method^32^. Seven-weeks-old C57BL/6 mice were sacrificed via decapitation and the skulls opened longitudinally along the midline. The hippocampus was dissected and cut into 400 μm cross-sections using a McIlwain tissue cutter (Ted Pella, Inc., Redding, CA, USA). The hippocampal slice was transferred to a dish containing the dissection medium and carefully separated with a pair of sterile spatulas using a dissecting microscope. Only intact-shaped sections were transferred to 0.4 μm MilliCell cell culture inserts (Millipore, Billerica, MA, USA) and deposited in 6-well plates. Four to six slices were placed on each insert and maintained in 1 mL of serum-based media consisting of 50% MEM-Hank’s medium, 25% horse serum, 25% HBSS, 5 mg/mL D-glucose, 50 mM HEPES, 2 mM L-glutamine and 1% antibiotic/antimycotic (all obtained from GIBCO Life Technologies). During the experiment, OHCs were maintained at 37 °C in 5% CO_2_, irradiated with LEDs at a wavelength of 660 nm for 24 h, and then used for histology and RNA extraction.

### Reverse transcription-polymerase chain reaction (RT-PCR) and quantitative PCR (qPCR)

Total RNA was prepared from ~100 mg of hippocampal tissue, which was dissolved in 1 mL TRIzol® Reagent and homogenized. Total RNA was quantified by spectrophotometer at 260 nm using NanoDrop 2000 (ThermoFisher, CA, USA), and RNA quality was checked by 1% agarose gel electrophoresis and ethidium-bromide staining. RNA samples were stored at −80 °C until use. cDNA was amplified with the following primers: *BDNF*, forward 5′-GAC AAG GCA ACT TGG CCT AC-3′ and reverse 5′-CCT GTC ACA CAC GCT CAG CTC-3′; and glyceraldehyde 3-phosphate dehydrogenase (*GAPDH*), forward 5′-ACA TTG TTG CCA TCA ACG AC-3′ and reverse 5′-ACG CCA GTA GAC TCC ACG AC-3′^33^. Amplified products were detected using 1% agarose gel by Gel Doc XR System (Bio-Rad, Hercules, CA, USA). Band densities were calculated using Image Lab software (5.0 version; Bio-Rad).

For qPCR, template cDNA was mixed with SYBR Green Master Mix containing Taq, dNTPs (Qiagen), and forward and reverse primers. The primers used were: *BDNF*, forward 5′- CGA CAT CAC TGG CTG ACA CT-3′ and reverse 5′- CAA GTC CGC GTC CTT ATG GT-3′; glutathione peroxidase (*GPx*), forward 5′-TCA CCA ACG TGG CCT CGC AAT G-3′ and reverse 5′-CCT TGA TTT CTT GAT TAC TTC CTG GCT CCT G-3′; superoxide dismutase 1 (*SOD1*), forward 5′-GGG TTC CAC GTC CAT CAG TAT-3′ and reverse 5′-GCG GCT CCC AGC ATT TC-3′; glutathione reductase (*GR*), forward 5′-TGC GTG AAT GTT GGA TGT GTA CCC-3′ and reverse 5′-CCG GCA TTC TCC AGT TCC TCG-3′; and b-*GAPDH* forward 5′-TGG GGT GAG GCC GGT GCT GAG TAT-3′, reverse 5′-CAT TGG GGG TAG GAA CAC GGA AGG-3′^34^. All experiments were performed in triplicate. After normalization for GAPDH expression levels, gene expression levels were analyzed. Analysis of relative gene expression data using real-time quantitative PCR and the 2^−ΔΔCq^ method^35^.

### Western blot analysis

Protein expression levels were assessed by western blot analysis. Cells were lysed and centrifuged at 16,000 g. Then, the supernatant was electrophoresed on 12% SDS-PAGE and transferred to a nitrocellulose membrane. Membranes were detected with primary antibody and horseradish peroxidase (HRP)-conjugated antibody after blocking for 1 h with 5% skim milk in Tween (0.05%)/PBS. The immunoreactive bands were visualized using West Femto Chemi-luminescent substrate (Thermo). Protein levels were normalized to β-actin expression using ChemiDOC™ XRS+ (Bio-Rad, Hercules, CA, USA).

### Immunohistochemistry

The hippocampus was fixed with 10% paraformaldehyde in 0.1 M PBS (pH 7.4) and embedded in paraffin, as previously described. Tissue sections were stained with hematoxylin-eosin (H&E), to assess the general tissue morphology, and subjected to immunohistochemistry^36^. Paraffin blocks were cut into 4~6-μm sections and mounted on glass slides. Sections were deparaffinized by treatment with xylene and serial dilutions of ethanol, stained with H&E, and then immunostained for p-ERK, p-CREB, and BDNF, to label cells that had migrated into the hippocampus. All slides were incubated in 0.3% H_2_O_2_ in methanol overnight at room temperature to quench endogenous peroxidase activity. Immunostaining was performed overnight at 4 °C with primary antibodies diluted at 1:200~1000 with 1% bovine serum albumin (BSA) in PBS. The slides were then incubated with an HRP-conjugated secondary antibody diluted at 1:500 in 5% BSA in PBS, at 37 °C for 1 h. The slides were also stained with 1% Schiff’s reagent and stained with Mayer’s hematoxylin for 5 min at room temperature. The ratio of tissue immunostaining positive cells is compared to the expression rate of the control results by the third repeat experiments.

### Statistical analysis

Data were presented as means ± SD. Statistical significance was determined by Student’s t-test for independent methods using Microsoft Excel. Statistical significance was set at 0.05.

### Ethics approval

All experiments were approved by the Ethic Committee of Keimyung University (approval number: KM-2017-43R2).

## Data Availability

All data and materials used in this study are available upon reasonable request.
